# Inaccurate Blood Pressure Readings in the Intensive Care Unit: An Observational Study

**DOI:** 10.7759/cureus.3716

**Published:** 2018-12-11

**Authors:** Brian Tse, Mark A Burbridge, Richard A Jaffe, John Brock-Utne

**Affiliations:** 1 Anesthesiology, Stanford University Medical Center, Stanford, USA

**Keywords:** cerebral perfusion, intensive care, arterial line

## Abstract

Measuring and monitoring cerebral perfusion pressure (CPP) is important in the management of patients with certain neurological conditions. To accurately reflect blood pressure at the circle of Willis, the arterial line transducer should be leveled at the tragus. This study measured the relative distance of the transducer to the tragus in 100 intensive care unit (ICU) patients in the mixed ICU at our institution, of which 44 patients had a pressure-sensitive neurological diagnosis. For neurological patients, the average distance was 10.9 cm and for non-neurological patients, the average distance was 11.4 cm (p-value: 0.60). This suggests that the arterial line transducer was leveled at approximately the same level regardless of pathology, potentially leading to falsely elevated CPP readings in patients with pressure-sensitive neurological pathology.

## Introduction

The arterial line is a critically important monitoring tool used in the operating room and intensive care unit (ICU) in order to obtain a continuous measurement of arterial blood pressure (ABP) and its waveform. Regardless of the location of the intra-arterial cannula, the vertical height of the pressure transducer measures the ABP at that vertical point of the body [1]. In most general ICUs, ABP transducers are leveled at the phlebostatic axis, the reference point for the right atrium, using the mid-axillary line at the level of the fourth intercostal space as external landmarks [2].

In patients with certain neurological conditions, the cerebral perfusion pressure (CPP) is an important parameter that directly correlates with cerebral blood flow, and therefore brain perfusion. Its measurement in clinical practice is calculated by the difference between mean arterial pressure (MAP) and intracranial pressure (ICP), or central venous pressure (CVP), if higher [3]. In order to manage CPP for various clinical scenarios, the accurate measurement of MAP is essential. Although many international guidelines have published and studied target values for CPP, none have specified how to measure the MAP for the CPP calculation until 2015, when the Neuroanaesthesia Society of Great Britain and Ireland (NASGBI) and Society of British Neurological Surgeons (SBNS) issued a joint position statement. They recommend that the arterial transducer be leveled at the tragus of the ear or external auditory meatus, which corresponds to the level of the middle cranial fossa and circle of Willis [4]. They also recommend the arterial transducer be re-positioned, to remain level with the tragus, after changes in head elevation.

In the mixed ICU of our quaternary care academic medical center, nurses are trained in maintenance of arterial lines and are responsible for the transducer height. It has been observed that the level of the transducer is transduced at the phlebostatic axis, even when CPP is of importance because no protocol exists to make height adjustments in patients who have pressure-sensitive neurological conditions. The goal of this study is to measure the relative height of arterial line transducers compared to the tragus.

## Materials and methods

This study started as a quality improvement project and was reviewed by the local Institutional Review Board (IRB) and the requirement for written informed consent was waived by the IRB. Patients included in this observational study were admitted to the Stanford University Hospital Medical and Surgical ICU between September 2017 and October 2017. This is a mixed ICU that has no physical division based on patient pathology, and does not have a dedicated neurosciences ICU. The inclusion criterion is the active measurement of ABP from an arterial line. The eligible patients were identified via electronic medical record (Epic Systems, Madison, WI). Each patient was evaluated at the bedside, where the height of the arterial line transducer relative to the tragus was measured as well as the degree of elevation of the head of bed position.

The ABP transducer height was measured using a string line level that originated from the patient’s tragus and extended to the pole of the ABP transducer. The height difference from the level of the tragus on the pole to the transducer was measured with a ruler and recorded. The head of bed position, indicated in degrees on the hospital bed, was recorded as well.

Each patient’s primary ICU diagnosis and active secondary diagnoses were collected and categorized as a neurological diagnosis or not. The neurological diagnoses included any neurological surgery (tumor resection, aneurysm clipping, vascular malformation resection), ischemic stroke, intracranial hemorrhage, hypoxic brain injury, traumatic brain injury (TBI), and/or encephalitis/meningitis. Basic descriptive statistics and a student’s *t*-test between the transducer height of the two groups were appropriately calculated.

## Results

**Figure 1:**
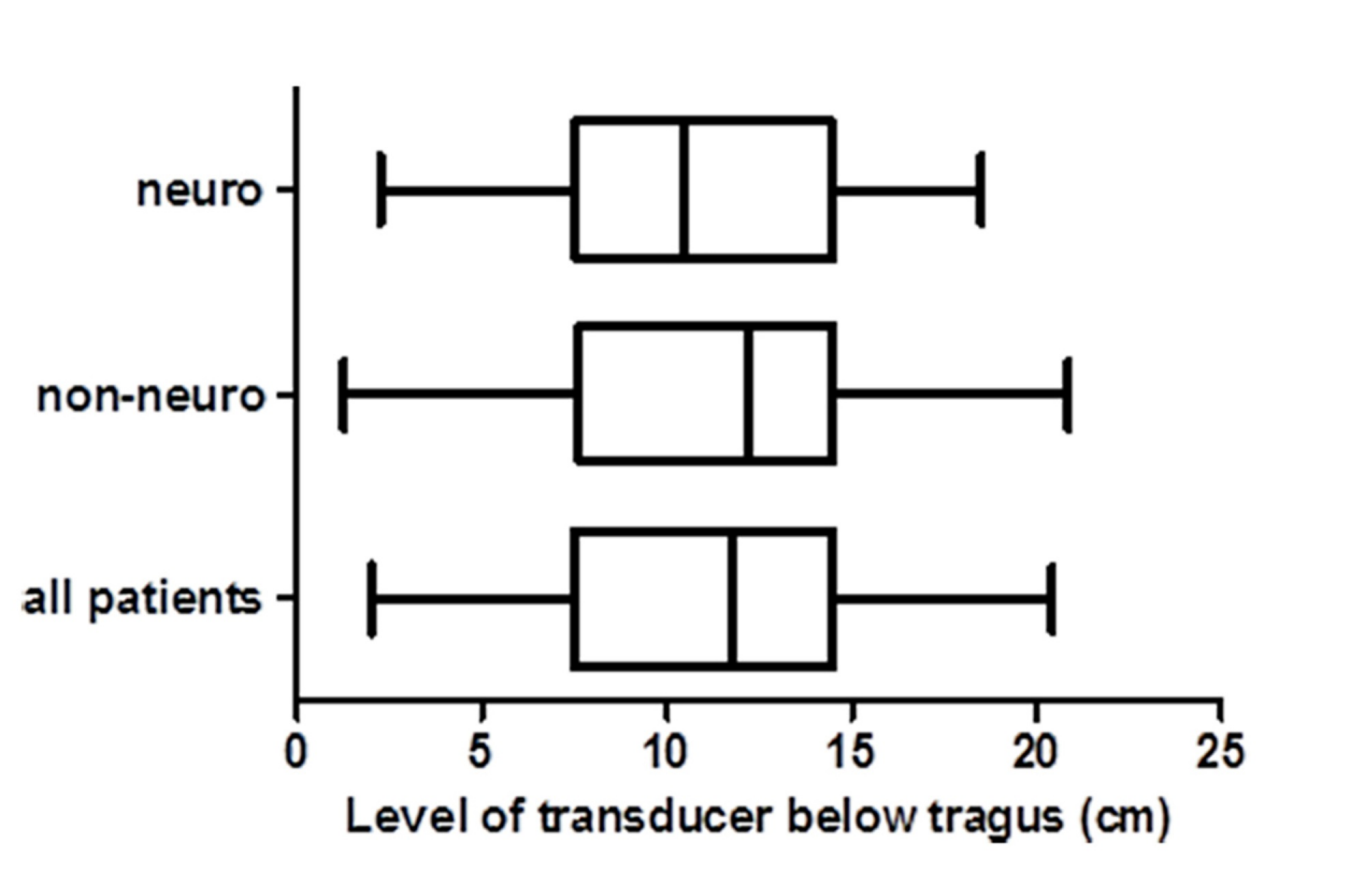
Box plots of transducer distances. Box plots of the distance of the transducer below tragus for patients with neurological diagnoses, non-neurological diagnoses, and for all patients.

Data from 100 patients with arterial lines were collected in the month-long observational period. There were 44 patients with neurological diagnoses and 56 in the non-neurological group. The average height of the transducer for all patients was 11.2 cm below the tragus. For neurological patients, the average height was 10.9 cm below the tragus and for non-neurological patients, the average height was 11.4 cm below the tragus. The difference in height between the two groups was 0.44 cm with a p-value of 0.60 (Figure 1).

**Figure 2:**
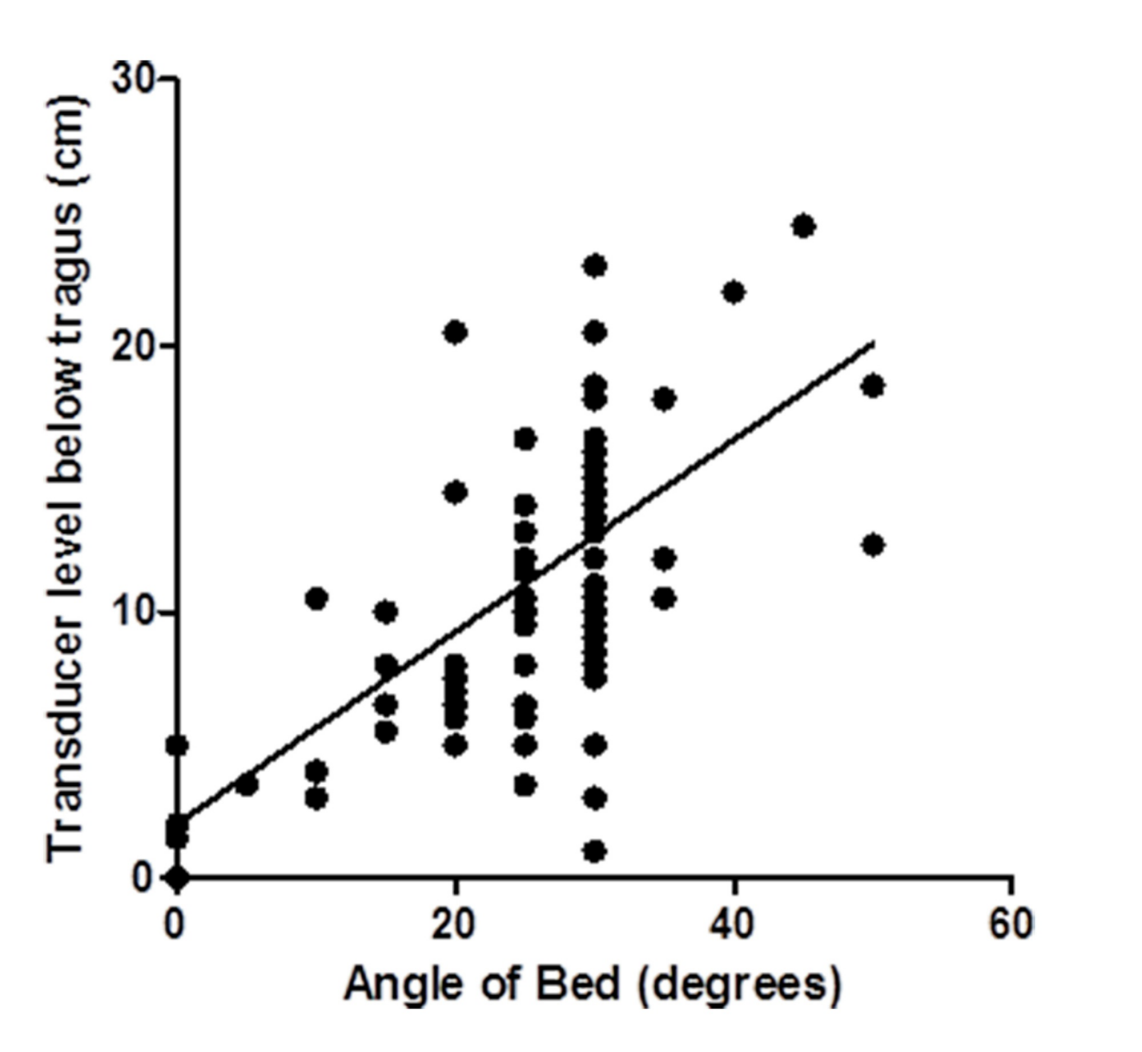
Transducer height relative to bed angle. Scatter plot of the transducer level and the angle of the bed with least squares regression line for all patients.

A scatter plot of the distance of the transducer below the tragus to the angle of the bed shows a positive correlation, with a Pearson’s *r* value of 0.65. This indicates that in general, a higher angle of the head of bed correlates with a greater distance of the arterial line transducer below the tragus (Figure 2).

## Discussion

This observational study showed that arterial line transducers in our ICU are leveled at approximately the same height below the tragus in patients with and without pressure-sensitive neurological pathologies. It also showed a negative correlation between the angle of the head of the bed and the distance of the transducer below the tragus. This suggests that at our institution, the transducer is leveled at the phlebostatic axis for all ICU patients, regardless of their diagnosis. It also demonstrates that as the angle of the head of the bed is increased, the discrepancy between MAP and CPP increases presumably because the arterial line transducer is not re-adjusted, and remains at the phlebostatic axis.

The clinical implication of this discrepancy is an inaccurate MAP, and therefore CPP reading, when the patient’s head is elevated. For instance, for a vertical distance of 10 cm, the corresponding change in MAP is approximately 7.5 mmHg. Using this study’s finding of an average distance of the transducer being 11 cm below the tragus, that equates to a MAP that should be 8 mmHg lower, which decreases the CPP measurement by 8 mmHg. For critically ill patients, this could potentially result in cerebral hypoperfusion and ischemia. It could also represent a critical issue in patients with vasocclusive disease such as Moyamoya disease whose cerebral perfusion is dependent on accurate MAP parameters at the risk of inducing transient ischemic attacks, strokes, or death. Moreover, patients who experience intra-operative neuromonitoring changes reflecting impending neurological compromise must often be maintained in a strict range of accurate MAPs [5-6].

We acknowledge this study is an observational study with no clinical outcomes measured. The validity of CPP directed management for TBI is also debatable, given the lack of level 1 evidence supporting it [7]. However, this could in part be due to the lack of consistency amongst studies attributed to the lack of standardization of transducer prior to the NASGBI and SBNS guidelines. Moreover, we also acknowledge that our patients may also have other clinical indications for ABP monitoring at the phlebostatic axis that may necessitate an additional transducer or arterial cannulation.

## Conclusions

In conclusion, our study shows that without placing the arterial line transducer at the tragus to better approximate CPP, major discrepancies can result, which is exacerbated by increasing the angle of the patient’s bed. We are currently engaged in discussions with our ICU leadership to educate and protocolize moving the height the ICU transducer to the tragus for indicated neurological pathologies, and would recommend other such ICUs to do the same.
